# Supplementary material for: Is the rise in childhood obesity rates leading to an increase in hospitalizations due to dengue?
Source: PLoS Negl Trop Dis. 2024 Jun 27;18(6):e0012248. doi: 10.1371/journal.pntd.0012248 (PMC11210816; doi:10.1371/journal.pntd.0012248)
Supplement: S1 Table — (DOCX) [file pntd.0012248.s001.docx]

|  | BMI Centile | | | | | |
| --- | --- | --- | --- | --- | --- | --- |
| District | <3rd | 3rd to 15th | 15th to 50th | 50th to 85th | 85th to 97th | >97th |
|  | N (%) | N (%) | N (%) | N (%) | N (%) | N (%) |
| Trinco  (N=236) | 40 (16.95%) | 47 (19.92%) | 62 (26.27%) | 49 (20.76%) | 23  (9.75%) | 15 (6.36%) |
| Polonnaruwa (N=231) | 62 (26.84%) | 35 (15.15%) | 62 (26.84%) | 43 (18.61%) | 19  (8.23%) | 10 (4.33%) |
| Kurunegala (N=757) | 179 (23.65%) | 143 (18.89%) | 201 (26.55%) | 128 (16.91%) | 71  (9.38%) | 35 (4.62%) |
| Jaffna  (N=297) | 48 (16.16%) | 52 (17.51%) | 92 (30.98%) | 59 (19.87%) | 27  (9.09%) | 19 (6.40%) |
| Ratnapura (N=465) | 126 (27.10%) | 101 (21.72%) | 135 (29.03%) | 66 (14.19%) | 26  (5.59%) | 11 (2.37%) |
| Kandy  (N=608) | 153 (25.16%) | 129 (21.22%) | 173 (28.45%) | 100 (16.45%) | 36  (5.92%) | 17 (2.80%) |
| Matara  (N=436) | 134 (30.73%) | 92 (21.10%) | 106 (24.31%) | 64 (14.68%) | 31  (7.11%) | 9  (2.06%) |
| Badulla  (N=478) | 85 (17.78%) | 130 (27.20%) | 145 (30.33%) | 72 (15.06%) | 27  (5.65%) | 19 (3.97%) |
| Gampaha (N=1274) | 230 (18.05%) | 210 (16.48%) | 322 (25.27%) | 281 (22.06%) | 151 (11.85%) | 80 (6.28%) |
| Total  (N=4782) | 1057 (22.10%) | 939 (19.64%) | 1298 (27.14%) | 862 (18.03%) | 411 (8.59%) | 215 (4.50%) |

**S1 Table: Percentages of children in different BMI centile categories for each of the 9 districts sampled and islandwide**
