# Supplementary material for: Is the rise in childhood obesity rates leading to an increase in hospitalizations due to dengue?
Source: PLoS Negl Trop Dis. 2024 Jun 27;18(6):e0012248. doi: 10.1371/journal.pntd.0012248 (PMC11210816; doi:10.1371/journal.pntd.0012248)
Supplement: S2 Table — (DOCX) [file pntd.0012248.s002.docx]

| Urbanicity | Total N=4782(%) | Dengue Seropositive N (%) | Hospitalisation Rates for Dengue in Dengue Seropositive Children N (%) |
| --- | --- | --- | --- |
| Urban | 840  (17.57%) | 293  (34.88%) | 32  (10.92%) |
| Rural | 3772 (78.88%) | 841  (22.30%) | 83  (9.87%) |
| Estate | 170  (3.55%) | 18  (10.59%) | 0  (0.00%) |

**S2 Table: Dengue seropositivity rates and hospitalisation rates for dengue in dengue seropositive children in urban, rural and estate areas islandwide.**
