# Supplementary material for: Is the rise in childhood obesity rates leading to an increase in hospitalizations due to dengue?
Source: PLoS Negl Trop Dis. 2024 Jun 27;18(6):e0012248. doi: 10.1371/journal.pntd.0012248 (PMC11210816; doi:10.1371/journal.pntd.0012248)

**Binary Logistic Regression Model summary – Demographic variables and BMI**


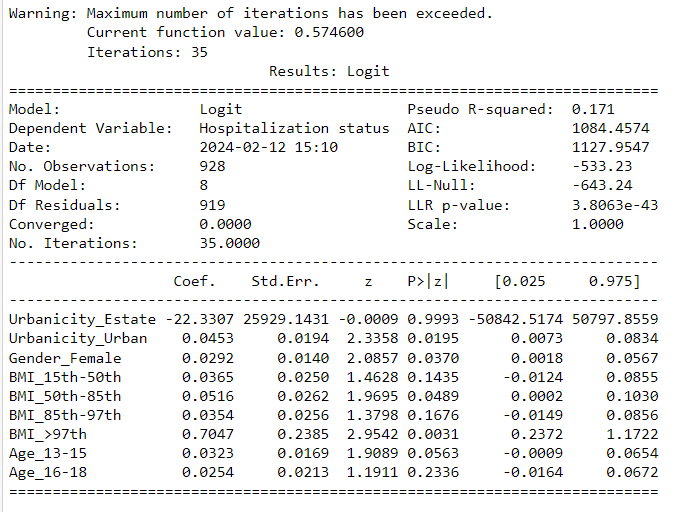


**Fitted** **Binary Logistic Regression Model**


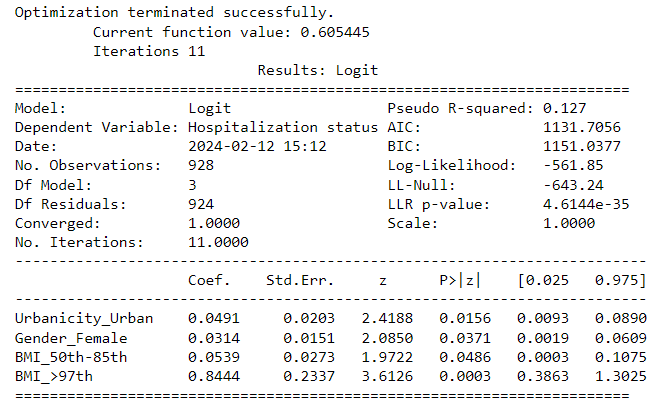


Odd ratio for the fitted model


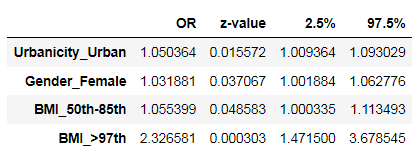

Supplement: S1 Data — (DOCX) [file pntd.0012248.s003.docx]
