# Supplementary material for: Is the rise in childhood obesity rates leading to an increase in hospitalizations due to dengue?
Source: PLoS Negl Trop Dis. 2024 Jun 27;18(6):e0012248. doi: 10.1371/journal.pntd.0012248 (PMC11210816; doi:10.1371/journal.pntd.0012248)
Supplement: Attachment — Submitted filename: Answer to review comments 21.05.2024.docx [file pntd.0012248.s006.docx]

21^st^ May 2024

Dr. Amy C. Morrison, PhD,

Section Editor,

PLOS Neglected Tropical Diseases

Dr. Andrea Marzi,

Section Editor,

PLOS Neglected Tropical Diseases

Dear Dr. Morrison and Dr. Marzi,

**Submission of manuscript “Is the rise in childhood obesity rates leading to an increase in hospitalizations due to dengue?”**

We wish to thank the reviewers for carefully going through our manuscript and the helpful suggestions they have made. We have incorporated all these changes in the revised version and have addressed each of those raised by the reviewers in the cover letter. We have highlighted the changes in the revised manuscript.

**Reviewer 1**

1. **Line 197: Worth making it clear to the reader what “imbalanced data” is being referred to here.**

**Response:** We apologize for the lack of clarity. We have amended the revised manuscript as follows: The associations between BMI, urbanicity socio-demographic factors to hospitalization status for dengue, was analyzed with the Binary Logistic Regression Model (please see supplementary data of this model) and implemented using the Synthetic minority oversampling technique (SMOTE) to recover the imbalanced data. The data was considered to be imbalanced as the number of hospitalized children were far fewer than the children who were not hospitalized . Therefore, in order to develop the best performing model, SMOTE model was implemented.

We believed it was best to use the resampling technique to handle such data where we have used a synthetic minority over sampling technique (SMOTE) to balance the dataset.

2. **The authors no present a logistic regression as requested. The reason I had originally requested this was so that co-morbidities could be included in that model. The eligibility excludes children with co-morbidities. I think this is fine, but I would make it clear in the description of the model within the methods, that co-morbidities were not assessed in the model as the children were all previously healthy (apart from varying BMIs).**

**Response:** We thank the reviewer for this suggestion. Indeed, none of the children had comorbidities and was previously healthy. The Binary Logistic Regression model was fitted taking into consideration the demographic variables and BMI categories only. We have indicated this in the methods in the revised version of the manuscript.

3. **The authors should present logistic regression summary tables, ideally in the main manuscript but could be in supplement.**

**Response:** We thank the reviewer for this suggestion, and we have included a summary of the model as supplementary data.

4. **I am unclear whether the results of “Urbanicity and risk of hospitalization” are derived from the same logistic regression analysis or a separate one. Could this be made clearer? The model summary tables will help resolve this. The methods mention 4. Spearman rank correlation coefficient, but where are these presented in the results? I mentioned this previously, but perhaps I wasn’t clear.**

**Response:** We apologize for the lack of clarity**.** Urbanicity was considered in the fitted regression model, and we have included this fitted model summary supplementary data in the revised version of the manuscript. We have removed the accidental inclusion of the portion on Spearman rank correlation coefficient in the revised version of the manuscript.

5. **Line 198: this is not a predictive model. I would put a fullstop after model and lose the rest of the sentence.**

**Response:** We thank the reviewers for this suggestion and have amended the manuscript as advised.

6. **Line 258: The authors mention a single weakness, “However, as we only assessed anthropometric measurements at the time of recruitment”. There are several other weaknesses, for example this is a retrospective and observational study. I would like to see a slightly deeper look at these. Having said this, one of the great strengths of this study, to me, are the innovative (albeit limited) methods. This approach does not incur the same time and resource costs as a prospective study and therefore I think this positive point is also worth mentioning.**

**Response:** We thank the reviewer for these positive comments and also highlighting further weaknesses. We have included this in the revised version of the manuscript.

7. **Finally, I think it is worth noting that** **only 12/115 hospitalized people were actually in the >97^th^ BMI centile group. I think this statement could be fairly easily integrated into the discussion. I personally think it helps contextualize the findings and reminds us of the importance of non-obesity related factors associated with hospitalisation. The exclusion of individuals with co-morbidities limits the ability of this study to compare the relative importance of obesity and other co-morbid factors.**

**Response:** We thank the reviewer for this suggestion, and we have included these sentences in the discussion. However, we did not exclude any children with co-morbidities as children did not have any comorbidities.

8. **Abstract Line 48: Clearly state the aim in the abstract**

**Response:** We thank the reviewer for this comment and have amended this in the revised version of the manuscript.

9. **Abstract Line 54: BMI should come directly after index. I don’t think you mean BMI= “Body Mass Index Centile”.**

**Response:** We thank the reviewer for pointing this out and have corrected this mistake.

10. **Abstract Line 58: Missing “Result” subheading**

**Response:** We thank the reviewer for pointing this out and have corrected this mistake.

11. **Abstract Line 60: Missing “regression” after logistic**

**Response:** We thank the reviewer for pointing this out and have corrected this mistake.

12. **Abstract Line 63: I think it should be “significantly associated with hospitalisation” not “hospitalisation rates”. This was a patient level analysis.**

**Response:** We appreciate the reviewer feedback and have amended the manuscript as advised.

13. **Author Summary: this is text recycled from manuscript and should be more aimed at the general public.**

**Response:** We thank the reviewer for this comment and have amended this in the revised version of the manuscript.

14. **Introduction Line 97: 390 starts the sentence, perhaps this should be written out.**

**Response:** We thank the reviewer for this comment and have written it out as suggested.

15. **Introduction Line 99 (and others): Is use of colons better replaced by the word “in”. e.g 1 in 4 rather than 1:4.**

**Response:** We thank the reviewer for this comment and have replaced colons with the word “in” in all instances of use.

16. **Introduction Line 131: “with dengue” is unnecessary.**

**Response:** We appreciate the feedback and have removed the words from the text.

17. **Introduction Line 132: is a bit muddled.**

**Response:** We thank the reviewer for pointing this out. We have amended this in the revised version of the manuscript.

18. **Introduction Line 145: Inconsistent use of digits and text for numbers. Here “nine” is used. In other places numbers are used e.g line 147. Good to check the whole manuscript.**

**Response:** We thank the reviewer for this suggestion. We have ensured that all text has been converted to digits across the manuscript.

19. **Introduction Line 150: What is an “estate” area?**

**Response:** We apologize for not describing this term and done so in the revised version. Estate areas are communities living in tea estates in the central highlands.

20. **Methods Line 141-2: should be “between the ages of 10 *and* 18” not “10 *to* 18”.**

**Response:** We thank the reviewer for this suggestion and have replaced the word as advised.

21. **Methods Line 162: ethics statement should be moved to the beginning or end of the methods.**

**Response:** We thank the reviewer for this comment. The ethics statement has been shifted to the end of the methods section.

22. **Methods Line 187: should be “and *to* implement models” not “and implement models”.**

**Response:** We thank the reviewer for this comment and have inserted the word “to” as advised.

23. **Results Line 210: “and age-stratified seroprevalence rates of each of the districts, and seropositivity rates based on urbanicity has been previously described for this cohort [15]”. Not sure why this is in the results.**

**Response:** We have added this sentence in the results, as we use the data to generate table 1.

24. **Results Line 215: starts with a number.**

**Response:** We thank the reviewer for this comment and have restructured the sentence to mitigate this issue.

25. **Results Line 222-3: Table 2 doesn’t test the significance of this difference, there are no p-values or confidence intervals presented.**

**Response:** We thank the reviewer for this comment and have inserted the relevant p-value immediately after the statement in the manuscript.

26. **Results Line 227: should be “logistic regression” not just “logistic”.**

**Response:** We thank the reviewer for this comment and have inserted the word as advised.

27. **Results Line 229: “was significantly associated with hospitalization rates when compared to children in other BMI categories”. It should state was significantly associated with hospitalization not “hospitalization rates” as this is a patient level analysis. Although, we need the model summary tables to be clear about this.**

**Response:** We thank the reviewer for this comment and have removed the word “rates” as advised.

28. **Discussion Line 255: first sentence needs to be a little clearer. “…when infected with DENV…” I don’t think is grammatically correct.**

**Response:** We aplogise for the lack of clarity, which we have corrected in the revised version.

29. **Discussion Line 258: should be “…twice as likely…” not “…twice as more likely…”**

**Response:** We thank the reviewer for this comment. We have removed the word from the manuscript.

30. **Discussion Line 270: “…steady rise in BMIs…” perhaps “…steady rise in the BMI of the population in…” or something like that.**

**Response:** We thank the reviewer for this comment and have restructured the sentence as suggested.

31. **Introduction Line 106: “However, if host factors such as the presence of comorbidities increase the likelihood of symptomatic infection leading to hospitalizations has not been studied”. I don’t think this is true. This paper does do this:** <https://www.sciencedirect.com/science/article/pii/S1201971221006172#sec0006>. **While this study does not discuss obesity it does look at host factors associated with hospitalisation, which is the claim made by the authors. I don’t think it is essential that this has never been studied for this paper to be valuable and I would strongly recommend removing concrete statements like this. I would rephrase it to say e.g “there is limited information on…”.**

**Response:** We thank the reviewer for pointing this out and have revised the introduction and abstract accordingly.

32. **Introduction Line 131: “Although obesity is a known risk factor for severe dengue in hospitalized patients with dengue [13, 14], whether obesity associates with an increase in hospitalizations has not been studied”. The fact that it has never been studied is not a sufficient justification for doing the study. I think you just need an additional sentence here (or at the end of paragraph 1) which states the extra value of this study over studies in hospitalized patients, there are several. This is what I was trying to say in my first review, apologies if it wasn’t clear.**

**Response:** We thank the reviewer for this suggestion and included this in the revised version of the manuscript.

Thank you for considering our manuscript with PLOS Neglected Tropical Diseases.

Yours Sincerely,

Prof. Neelika Malavige
