# Supplementary material for: Is the rise in childhood obesity rates leading to an increase in hospitalizations due to dengue?
Source: PLoS Negl Trop Dis. 2024 Jun 27;18(6):e0012248. doi: 10.1371/journal.pntd.0012248 (PMC11210816; doi:10.1371/journal.pntd.0012248)
Supplement: Attachment — Submitted filename: Reponses to Reviewer Comments 18.03.2024.docx [file pntd.0012248.s005.docx]

18^th^ March 2024

Dr. Amy C. Morrison, PhD,

Section Editor,

PLOS Neglected Tropical Diseases

Dr. Andrea Marzi,

Section Editor,

PLOS Neglected Tropical Diseases

Dear Dr. Morrison and Dr. Marzi,

**Submission of manuscript “Are the rise in childhood obesity rates leading an increase in hospitalizations due to dengue?”**

We wish to thank the reviewers for carefully going through our manuscript and the helpful suggestions they have made. We have incorporated all these changes in the revised version and have addressed each of those raised by the reviewers in the cover letter. We have highlighted the changes in the revised manuscript.

**Overall Comments**

1. **A major issue for me was a lack of details on the retrospective analysis.  It is important to know how far back (years) were "hospitalized" cases included: 10 years, 5 years, 2 years, 1 year.**

**Response:** We thank the editors for this very relevant question. During the serosurvey, parents/guardians were asked to come to schools with all previous medical records. In Sri Lanka, when someone is hospitalized, they receive a diagnostic card when the leave the hospital, which states their diagnosis (please see an example attached). Therefore, hospitalizations due to dengue were only considered if the parents/guardians had evidence to show that the children had been hospitalized due to dengue. Therefore, any hospitalization due to dengue in the past was considered in the analysis. This is of course a limiting factor and we have mentioned all this, including the limitation in the revised version of the manuscript.

**2. I do not need to say that your IgG ELISA probably indicates exposure in the past but is not necessarily associated with the hospitalization record.  Was there definitive laboratory confirmation of a dengue case at the time of admission and if so how.**

**Response:** Sri Lanka, as many other countries in the world, do not carry out confirmatory testing for dengue in patients admitted to hospital. The diagnosis of dengue is made based on clinical features, which includes thrombocytopenia and other relevant clinical and laboratory evidence. We agree with the editors and the reviewers that this indeed is a limitation. Nevertheless, we believe the manuscript highlights valuable data on obesity and risk of hospitalization. Also, we have included all the additional analysis suggested by the reviewers, using different statistical methods and still find strong associations.

**3. Could you distinguish between patients who sought care but were managed as outpatients -- that is your IgG positive children without hospitalization should be called non-hospitalized, they could have had a range of symptoms at home including inapparent infections.**

**Response:** We thank the editors for this very important suggestion and changed the manuscript wording as suggested.

**4. Again the concerns that BMI and data collected during the cross-sectional survey would be consistent with that observed in the past when the child had dengue.**

**Response**: As mentioned by the editors and reviewers, this indeed is a major limitation, which we have highlighted in the revised version of the manuscript.

**5. Also think the suggestions for a more sophisticated multivariate statistical approach is warranted.**

**Response:** We agree with this suggestion and have done this in the revised version of the manuscript.

**6. I appreciated the creative approach (retrospective approach) your research group took to examine the role of obesity in dengue severity.  If retrospective review of hospital records (not just parents records - unless they are complete), would provide make this a lot more convincing.**

**Response:** We apologise for the lack of clarity. Parents records were not used for the analysis. All details were only obtained from a diagnostic card issued by the hospital when the patient is discharged. We have attached a sample of a diagnostic card (de-identified), for the editors and reviewers to see, which is not for publication purposes. As shown, all details including clinical and laboratory details (including changes in platelet counts and other parameters) are mentioned in the diagnosis card.

**Reviewer 1**

**1. The study’s aim is well described in the last sentence of the introduction. However, the justification could be strengthened. What additional information will this study provide beyond the existing studies that show an association between obesity and severe disease in hospitalized patients? While I believe it does add value—for instance, this study utilizes a community-based denominator which might be more representative of the general population than hospitalized patients—there are other unique aspects that should be explicitly stated.**

**Response:** We thank the reviewer for this suggestion. As mentioned by the reviewer, there are existing studies showing that obesity is associated with more severe disease in hospitalized patients. However, there is no data showing that obesity increases the likelihood of hospitalization in acute dengue.

**2. I found that the methods section lacked the necessary detail to answer this question (is the study design appropriate to address the stated objectives?). For instance, were hospitalizations from any time in the past considered? How can you ensure that the current BMI centile accurately reflects the BMI at the time of admission? Additionally, there is no mention of adjustments for confounders, the most significant of which could be co-morbidities.**

**Response:** Again, we agree with the reviewer and apologize for not including details. We have included all this and the limitations in the revised version. In addition, we have explained the methods in the answers above to the editors.

**3. Dividing the population into 10-18 years and 19-20 years groups is a bit confusing due to the different approaches to scoring obesity. Is this segmentation necessary? If there's no way to apply the same scoring method across the entire age range, perhaps consider only including those aged <=18. The conclusions would remain consistent, and the sample size is still substantial with n=4,782 (1,152 sero-positive).**

**Response:** We agree with this suggestion and agree that it is confusing. Therefore, we have limited our analysis to children ≤ 18 years of age.

**4. A map of the study sites would be a beneficial addition.**

**Response:** We thank the reviewer for this suggestion and have included this in the revised version of the manuscript.

**5. I'm concerned about the absence of an adjusted analysis. There wasn't any adjustment for age, co-morbidities, or social class—all of which could act as confounders. Additionally, it's worth noting that obesity and these other factors vary over time, implying they might have differed at the time of admission. If an adjusted analysis isn't feasible, then this should be clearly highlighted as a limitation in the discussion section.**

**Response:** We thank the reviewer for this very important suggestion. We have renalysed the data according to the suggestions by the reviewer in the revised version of the manuscript.

**6. I don't see the following analyses described in the "statistical analysis" section in the results:
"As the data were not normally distributed, differences in means were compared using the Mann-Whitney U test (two-tailed), and the Kruskal-Wallis test was used to compare the differences of the antibody levels in the different districts, and in urban, rural, and estate sectors." OR" Spearman rank order correlation coefficient was used to evaluate the correlation between age and DENV-specific antibody levels (Panbio155 Units)."
Also, the statistical analysis plan doesn't present methods for the "Urbanicity and risk of hospitalization" section in the results.**

**Response:** Again, we agree with the reviewer and apologize for not including details. We have included all this in the revised version of the manuscript. GraphPad Prism version 9.5 and Jupyter Notebook (python IDE) was used for statistical analysis and implement models. As the data were not normally distributed, differences in means were compared using the Mann-Whitney U test (two tailed), and the Kruskal-Wallis test was used to compare the differences of the antibody levels in the different districts, and in urban, rural and estate sectors. Spearman rank order correlation coefficient was used to evaluate the correlation between the age and DENV-specific antibody levels (Panbio Units) and Chi Square tests or the Fisher’s exact test was used to determine the p value. The associations between BMI, urbanicity socio demographic factors and hospitalization status for dengue, was analysed with the Binary Logistic Regression Model and implemented Synthetic minority oversampling technique (SMOTE) to recover the imbalanced data in order to develop the best performing model to predict the risk of hospitalization due to these factors.

**7. The text results are sufficient, but some additional tables and figures would help clarify the findings. Table 1: A summary of the demographics of the population (traditional Table 1), presenting: Age, district, gender, seropositivity, urbanization, etc. Table 2: The current table 1 could be a new table 2.**

**Figure 1: A figure showing how the case hospitalization rate varies with BMI centile categorically. With BMI centile category on the X-axis and case hospitalization rate on the Y-axis.**

**Response:** We thank the reviewer for this very important suggestion. We have included the tables and figures as suggested by the reviewer in the revised version of the manuscript.

**8. There a significant lack of context and depth in the discussion.**

**Response:** We thank the reviewer for this comment and have included a more details discussion in the revised version.

**9. There a some reflection on current literature. However, what do prior studies highlight about other risk factors for hospitalization? How might these factors be related to obesity?**

**Response:** This is indeed a very good question and suggestion. We have included additional analysis and included a relevant discussion with what has been observed in past studies and data from additional analysis.

**10. It's noteworthy that, while obesity was associated with hospitalization, the vast majority of individuals aged <=18 who were hospitalized fell below the 97th BMI centile (103 out of 115). This observation deserves further elaboration. What implications does this finding have for your overarching conclusions? Further, it's intriguing that only the >97th BMI centile group showcased a heightened admission rate. Why wasn't there a gradational increase observed across the various BMI groups?**

**Response:** This is indeed an interesting observation and we believe it could be possibly due to extreme obesity associated with increased risk due to a dysfunctional immune response? We have included this in the revised version of the manuscript.

**11. There is no in-depth discussion of the limitations of this paper.**

**Response:** We aplogise for not including limitations, which is a major shortcoming. We have included the limitations.

**12. The authors do not significantly discuss how these data can be helpful to advance our understanding of the topic under study**

**Response:** We aplogise for the lack of discussion on this matter and included this in the discussion in the revised version.

**13. More detail could be provided on the public health relevance. How big a factor is obesity likely to be compared to other risk factors for admission/severity?**

**Response:** We thank the reviewer for this suggestion and discussed this in the revised version of the manuscript.

**14. I find some of the claims in the paper over stated. For example, in the abstract "Obesity appears to increase the risk of hospitalization in those with dengue.". This implies causation, however, this is an observational study and is assessing for an association. The title whilst pharased as a question is also potentially misleading. "Are the rise in childhood obesity rates leading an increase in hospitalizations due to dengue?". This study cannot answer this question as it is observational. I think the claim in the disucssion is much less controversial "the first study reporting that obesity is also associated with higher rates of hospitalization".**

**Response:** We agree with the reviewer and therefore, we have changed the wording of the abstract and elsewhere.

**15. Explaining in more detail how dengue severity was determined (in Methods). Were all hospitalisation included, regardless of how long ago. Were paired samples taken?**

**Response:** We thank the reviewer for this very relevant question. During the serosurvey, parents/guardians were asked to come to schools with all previous medical records. In Sri Lanka, when someone is hospitalized, they receive a diagnostic card when the leave the hospital, which states their diagnosis (please see attached example of a patient with dengue who also had a secondary bacterial infection as a complication). Therefore, hospitalizations due to dengue were only considered if the parents/guardians had evidence to show that the children had been hospitalized due to dengue. Therefore, any hospitalization due to dengue in the past was considered in the analysis. This is of course a limiting factor and we have mentioned all this, including the limitation in the revised version of the manuscript.

**Reviewer 2**

**1. The exposure is the body mass index centile (BMI) and the main outcome hospital admissions. However, in the introduction the authors suggest the potential association between obesity with the risk of symptomatic infection, a driver of hospitalizations, suggesting that obesity could be a risk factor to suffer a symptomatic dengue disease
Also, as end-point the title of the paragraph “determining past dengue disease severity” could be cause confusion about the research question of the study.**

**Response:** We apologise for the lack of clarity. We have rephrased these sentences for better clarity in the revised version of the manuscript.

**2. Dengue prognosis study require longitudinal design to obtain outcomes as hypotension (determined by age-specific) tachycardia, signs of circulatory insufficiency, any abnormal neurological sign, etc, etc.  In this case a cross-sectional will be an inappropriate study design to address this objective.**

**Response:** We completely agree with the reviewer that the most suitable study design would be a longitudinal prospective one. In Sri Lanka, when someone is hospitalized, they receive a diagnostic card when the leave the hospital, which states their diagnosis. Details regarding disease severity, treatment given, if fluid leakage was present, results of blood tests etc.. are all mentioned in this diagnosis card. We have attached a sample of a diagnostic card (de-identified), for the editors and reviewers to see, which is not for publication purposes. As shown, all details including clinical and laboratory details are mentioned in the diagnosis card.

**3. The authors should be including the estimates with 95% confidence interval to demonstrate the study has obtained accuracy results thanks to the sample size selected. All of us know the advantages of a confidence interval (rather than a P value) to evaluate research questions.**

**Response:** We thank the reviewer for this very important suggestion. We have included this in the revised version of the manuscript. The evaluation of the model done by cross validation and confusion matrix revealed in the test dataset and 89 observations predicted as true negatives and 38 observations predicted as true positives. 5 observations predicted as false positives and 54 observations predicted as false negatives. The accuracy of the binary logistic regression classifier is 68% and the receiver operating characteristic score is 0.60 which considered to be model is satisfied for the prediction of hospitality rate with the associated factors.

**4. The study was conducted during the COVID pandemic period.  When you evaluate clinical record, how to differentiate hospitalized Covid-19 from hospitalized dengue cases? any clinical definition?  RT-PCR, NS1, IgM test, other methods were conducting for dengue diagnosis? Are Outpatients considered inapparent dengue?**

**Response:** We thank the reviewer for this very relevant question. During the serosurvey, parents/guardians were asked to come to schools with all previous medical records. In Sri Lanka, when someone is hospitalized, they receive a diagnostic card when the leave the hospital, which states their diagnosis. Therefore, hospitalizations due to dengue were only considered if the parents/guardians had evidence to show that the children had been hospitalized due to dengue. Therefore, any hospitalization due to dengue in the past was considered in the analysis. This is of course a limiting factor and we have mentioned all this, including the limitation in the revised version of the manuscript. We have attached a sample of a diagnostic card (de-identified), for the editors and reviewers to see, which is not for publication purposes. As shown, all details including clinical and laboratory details are mentioned in the diagnosis card.

**5. Could you provide information about people who receive medical care and were not hospitalized?**

**Response:** We apologize for the lack of clarity. Those who were not hospitalized but had febrile episodes, could have had mild dengue. However, in Sri Lanka (similar to almost all dengue endemic countries), dengue confirmatory tests are not done in those presenting with febrile illnesses to outpatient settings due to non-availability of such tests in public hospitals. All febrile illnesses are monitored as potential dengue infections, but there are no records kept by anyone of such febrile illnesses. It would be difficult to collect such data in an island wide cross-sectional study and we have mentioned this as a limitation in the revised version.

**6. The characteristics of the baseline of the study population should be informed with more detail.   Covariates as sex, age (maybe 10-14, 15-18, more than 18 years), the informant’s education level, any enrolled household member  having had a dengue illness diagnosed by a physician either at a hospital , anothers available.**

**Response:** We thank the reviewer for this very important suggestion. We have included the tables and figures as suggested by the reviewer in the revised version of the manuscript.

**7. I suggest compared, in bivariate analysis, the prevalence of seropositivity by categories of sociodemographic characteristics. These characteristics are important to appreciate the similarity between groups by covariate’s if not balanced or if the imbalance is not statistically adjusted, these characteristics can cause confounding and can bias study results. This requires adjusted analysis takes into account this baseline between groups that may influence the outcome.**

**Response:** We thank the reviewer for this very important suggestion. We have reanalyzed the data according to the suggestions by the reviewer in the revised version of the manuscript.

**8. I cannot identify an specific analysis plan in the manuscript. May be the prevalence ratios could be estimated from generalized estimating equations (GEE) with the Poisson distribution, using the robust sandwich estimate of the variance to account for intra-family correlations.**

**Response:** Thank you for this comment**.** We have not estimated the prevalence ratio as our objective is to estimate the risk of hospitalization associated with obesity and socio demographic factors.

**9. There are not description about the limitations of analysis. The characteristics of the baseline of the population study should be informed in detail to appreciate the differences between groups by covariate.**

**Response:** We aplogise for not including limitations, which is a major shortcoming. We have included the limitations and also the baseline characteristics in the revised version of the manuscript.

**10. The conclusions is supported by the data presented, but the information provided by the authors is limited. To evaluate the hypothesis and answer the research question is necessary to conduct a multivariate analysis to covariable adjustment and to estimate the potential confusion bias study results.**

**Response:** We thank the reviewer for this very important suggestion. We have renalysed the data according to the suggestions by the reviewer in the revised version of the manuscript and also done a more detailed discussion about limitations.

**11. Because the hypothesis is not novel, the background of this study should be supported with the addition of relevant evidence that has been provide for many authors. I suggest to review Kalayanarooj (Southeast Asian J Trop Med 2005), Kabra (Trans R Soc Trop Med Hyg 19999), Marón (Am J Trop Med Hyg 2010) and  Chen, CY., Obesity as a clinical predictor for severe manifestation of dengue: a systematic review and meta-analysis (BMC Infect Dis 23)**

**Response:** We thank the reviewer for these suggestions and included the above published reports in our discussion.

**Reviewer 3**

**1. Degree of associations between BMI, urbanicity and the risk of hospitalization, was expressed as the odds ratio (OR), there are other statistical methods that could be used to give it greater statistical power. Or use odd ratio adjusted**

**Response:** We thank the reviewer for this comment and included additional statistical analysis and data in the revised version of the manuscript.

**2. The number of hospitalized people is not very large; they should be very cautious when interpreting the results.**

**Response:** We apologize for not including limitations, and the baseline characteristics in the revised version of the manuscript.

**3. The authors analyze the urbanization variable and the risk of hospitalization. Although they did not find an association when analyzing this variable, it is not well described what health services care is like in urban and rural areas.**

**Response:** We have included all this additional analysis in the revised version of the manuscript and also described the standard of care for dengue in Sri Lanka, which is similar in rural and urban settings in the public sector hospitals. All the sub categories of the urbanization was considered in the model and mentioned the significant categories in the summary table with respect to the reference variable.

**4. The authors must always keep in mind that seroprevalence studies have limitations to affirm something, so their objectives and conclusions must be very specific but they do not mention limitations, including the use of IgG elisa for the confirmation of dengue infection is questionable given its cross reactions. These limitations need to be include in the discussion**

**Response:** We apologise for not including limitations, which is a major shortcoming. We have included all this in the revised version of the manuscript.

**5. The authors did not use or include or mention whether or not the patients had comorbidities and this information is also important to take into consideration and could impact the final outcome of the dengue episode for severity or not.**

**Response:** We thank the reviewer for this very important suggestion. We have included the clarification as suggested by the reviewer in the revised version of the manuscript.

**6. They use the history of previous hospitalization, this data was obtained from health facilities, being public records we always do not have 100% guarantee that this episode was severe, perhaps only somewhat mild but this depends on the care system, so this variable could confuse the results**

**Response:** We thank the reviewer for this very relevant question. During the serosurvey, parents/guardians were asked to come to schools with all previous medical records. In Sri Lanka, when someone is hospitalized, they receive a diagnostic card when the leave the hospital, which states their diagnosis. Therefore, hospitalizations due to dengue were only considered if the parents/guardians had evidence to show that the children had been hospitalized due to dengue. Therefore, any hospitalization due to dengue in the past was considered in the analysis. This is of course a limiting factor and we have mentioned all this, including the limitation in the revised version of the manuscript. We have attached a sample of a diagnostic card (de-identified), for the editors and reviewers to see, which is not for publication purposes. As shown, all details including clinical and laboratory details are mentioned in the diagnosis card.

Thank you for considering our manuscript with PLOS Neglected Tropical Diseases.

Yours Sincerely,

Prof. Neelika Malavige
